# miR-210-3p protects against osteoarthritis through inhibiting subchondral angiogenesis by targeting the expression of TGFBR1 and ID4

**DOI:** 10.3389/fimmu.2022.982278

**Published:** 2022-09-29

**Authors:** Han Tang, Wenrun Zhu, Lu Cao, Jin Zhang, Juncheng Li, Duan Ma, Changan Guo

**Affiliations:** ^1^ Department of Orthopedic Surgery, Zhongshan Hospital, Fudan University, Shanghai, China; ^2^ Key Laboratory of Metabolism and Molecular Medicine, Ministry of Education, Department of Biochemistry and Molecular Biology, School of Basic Medical Sciences, Fudan University, Shanghai, China

**Keywords:** osteoarthritis, miR-210-3p, angiogenesis, subchondral bone, adeno-associated virus

## Abstract

Excessive subchondral angiogenesis is a key pathological feature of osteoarthritis (OA), as it alters the balance of subchondral bone remodeling and causes progressive cartilage degradation. We previously found that miR-210-3p correlates negatively with angiogenesis, though the specific mechanism of miR-210-3p-related angiogenesis in subchondral bone during OA progression remains unclear. This study was conducted to identify the miR-210-3p-modulating subchondral angiogenesis mechanism in OA and investigate its therapeutic effect. We found that miR-210-3p expression correlated negatively with subchondral endomucin positive (Emcn^+^) vasculature in the knee joints of OA mice. miR-210-3p overexpression regulated the angiogenic ability of endothelial cells (ECs) under hypoxic conditions *in vitro*. Mechanistically, miR-210-3p inhibited ECs angiogenesis by suppressing transforming growth factor beta receptor 1 (TGFBR1) mRNA translation and degrading DNA-binding inhibitor 4 (ID4) mRNA. In addition, TGFBR1 downregulated the expression of ID4. Reduced ID4 levels led to a negative feedback regulation of TGFBR1, enhancing the inhibitory effect of miR-210-3p on angiogenesis. In OA mice, miR-210-3p overexpression in ECs *via* adeno-associated virus (AAV) alleviated cartilage degradation, suppressed the type 17 immune response and relieved symptoms by attenuating subchondral Emcn^+^ vasculature and subchondral bone remodeling. In conclusion, we identified a miR-210-3p/TGFBR1/ID4 axis in subchondral ECs that modulates OA progression *via* subchondral angiogenesis, representing a potential OA therapy target.

## Graphical Abstract

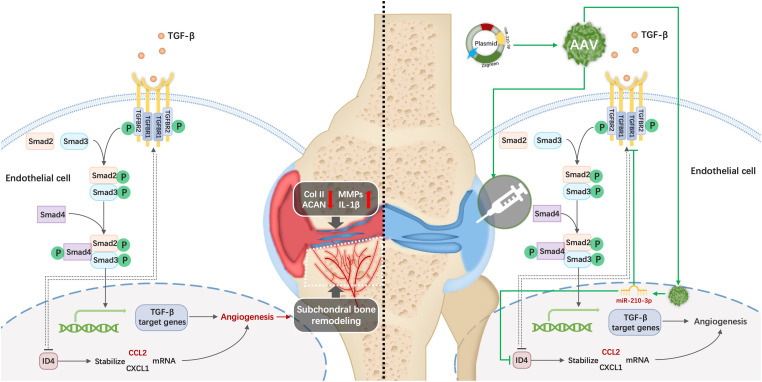

## Introduction

Osteoarthritis (OA) is a chronic degenerative disease that causes high morbidity and disability rates, endangers the health and quality of life of patients and increases medical care system burden and socioeconomic costs (1, 2). The pathogenic factors of OA include aging (3), mechanical alteration (4), dysfunction of synthesis and metabolism (5), inflammation (6) and immune abnormalities (7). All these complex factors result in joint tissue lesions mainly characterized by cartilage damage, subchondral bone remodeling and osteophyte formation (8–13). In recent years, substantial progress has been achieved in understanding the role of subchondral bone remodeling in the pathogenesis of OA (14–16). As subchondral bone provides mechanical stress support and is the main source of nutrients for cartilage, microenvironmental and structural changes in subchondral bone might affect cartilage metabolism directly or indirectly and are presumed to be one of the factors initiating OA (14). Specifically, aberrant angiogenesis in subchondral bone alters the balance of bone remodeling and results in sclerosis of subchondral bone in pre-OA lesions, which changes the mechanical stress characteristics of the subchondral bone and the nutrition supply pattern of cartilage and increases cartilage vulnerability (14, 17). In advanced OA, increased neurovascular coupling causes the most significant clinical symptom of OA, pain (18). Meanwhile, the subchondral vasculature gradually invades the cartilage through the tidemark and causes more serious degradation of the cartilage matrix (19). Thus, inhibition of subchondral angiogenesis is proposed to be crucial in preventing the pathological progression of OA, allowing cartilage to maintain a “healthy” state.

Bone is a particularly hypoxic tissue with pO_2_ levels ranging from less than 1% to 6% (20, 21), and the pO_2_ levels in the deep zone of articular cartilage are also less than 1% (22). Thus, vascular invasion from subchondral bone to cartilage occurs under hypoxic conditions *in vivo*. Notably, miR-210-3p, called the “master miRNA of hypoxia”, exerts multiple and complex effects on different diseases and tissues (23–25). We previously reported significantly increased expression of miR-210-3p in tissues with avascular necrosis of the femoral head (26), indicating that miR-210-3p may be negatively correlated with angiogenesis in subchondral bone. In particular, miR-210-3p manifests antiangiogenic properties in diseases with aberrant vascular formation. For example, miR-210-3p significantly inhibits the angiogenic ability of human retinal vascular endothelial cells by directly targeting insulin-like growth factor 2 (IGF2) in the retina (27). In preeclampsia, overexpression of miR-210-3p impairs extravillous trophoblast formation of endothelial-like networks (28). Therefore, we assume that the decrease in miR-210-3p expression may be attributed to angiogenesis in subchondral bone during the development of OA.

Here, we aimed to study the mechanism by which miR-210-3p affects subchondral angiogenesis and its therapeutic effect on OA. As a method to achieve our goal, we first overexpressed and inhibited miR-210-3p expression in endothelial cells (ECs) to determine its effect on the angiogenic ability of ECs *in vitro*. RNA sequencing and subsequent molecular experiments were implemented to determine the targets and downstream pathways of miR-210-3p. *In vivo*, we selectively overexpressed miR-210-3p in ECs of mice with anterior cruciate ligament transection (ACLT) induced OA to verify its role in the progression of OA using an adeno-associated virus (AAV) containing the tyrosine kinase with immunoglobulin like and EGF like domains 2 (TIE2) promotor (miR-210-3p/TIE2/AAV). We then performed CatWalk, microCT, histological staining, flow cytometry and immunochemistry experiments to evaluate the progression of OA. The regulatory mechanisms employed by miR-210-3p were also investigated in our study. We highlight a novel role of the miR-210-3p/transforming growth factor beta receptor 1 (TGFBR1)/DNA-binding inhibitor 4 (ID4) axis in subchondral ECs that modulates the progression of OA *via* subchondral angiogenesis, thereby suggesting a potential target for OA therapy.

## Materials and methods

### Cell culture and transduction

Human umbilical vein endothelial cells (HUVECs) were obtained from the Cell Bank of the Chinese Academy of Sciences (Shanghai, China). HUVECs were cultured in Dulbecco’s modified Eagle’s medium (BI, Israel) supplemented with 10% fetal bovine serum (Gibco, Grand Island, NY, USA), 50 U/ml penicillin and 50 μg/ml streptomycin in the presence of 5% CO2 and 1% O2 at 37°C in a humidified incubator. All culture media were renewed every 3 days. The miR-210-3p mimic, inhibitor and each negative control were synthesized by Ribobio (Guangzhou, China) and transfected into HUVECs using Lipo3000 (Invitrogen, Carlsbad, CA, USA) according to the manufacturer’s protocol. In addition, we constructed shRNAs to knock down the expression of TGFBR1 and ID4 using the pLKO.1-puro vector. Scrambled shRNA was used as shRNA negative control. Then, we packaged lentiviral particles in HEK293T cells using a packaging system including GAG, TAT, Rev, and VSVG vectors and constructed plasmids. We collected culture medium containing lentiviral particles at 48 and 72 hours and filtered it with a 0.45 μm filter. HUVECs at a 60-80% density were infected for 24 hours and selected with blasticidin, puromycin or neomycin for 7 days to establish overexpression or KD cell lines. The shRNA sequences are listed in **Table S1**.

### Transwell assay

HUVECs cultured under different intervention conditions were digested with 0.25% trypsin and adjusted to a density of 2 × 10^5^ cells/ml with serum-free DMEM. Then, 100 μL of the resuspended cells were added to the upper chamber of transwell plates (8 μm pore size) (Corning, NY, USA). Then, 600 μL of DMEM containing 10% FBS were added to the lower chamber. After 18 h of incubation at 37°C in a 5% CO2 incubator, the cells were fixed with methanol for 10 min and stained with a 0.1% crystal violet staining solution for 5 min. Next, cotton swabs were used to gently remove cells remaining on the top of the filter. Images of migrated cells were captured using an inverted microscope. Five visual fields were randomly chosen, and stained cells were counted by ImageJ software.

### EdU staining

An EdU staining kit was purchased from Ribobio (Guangzhou, China). According to the manual, we diluted the EdU solution at a ratio of 1000:1 with complete culture medium to reach a concentration of 50 μM. Then, we added 100 μL of the diluted EdU solution to each well and incubated the samples for 2 hours. After the incubation, we fixed the cells in each well with 4% paraformaldehyde for 30 min at room temperature, added 50 μL of 2 mg/mL glycine to each well, and incubated the samples for 5 minutes. Next, 100 μL of penetrant (0.5% Triton X-100 in PBS) were added to each well, and the samples were incubated for 10 minutes. Then, we applied Apollo^®^ 567 staining solution and Hoechst for EdU and nuclear staining, respectively. The reaction solution was incubated in the dark at room temperature for 30 minutes. Finally, each well was washed with 100 μL of PBS 1~3 times. Images were captured immediately using a fluorescence microscope after the staining was completed.

### Tube formation

Ninety-six-well plates were precoated with 50 μL of Matrigel (Corning, NY, USA) and incubated at 37°C for 30-60 minutes. A total of 50 μL of the HUVEC suspension (4×10^5^ cells/ml) was seeded in Matrigel-coated 96-well plates and incubated with 5% CO2 at 37°C in a humidified incubator for 6 hours. After the incubation, the formation of tube-like structures was assessed using a phase contrast microscope and ImageJ software.

### Western blotting

Adherent cells were digested with RIPA buffer (Biotime, Shanghai, China) containing PMSF (Yeasen, Shanghai, China) after two washes with ice-cold PBS. Twenty micrograms of total protein from each sample were separated on SDS–PAGE gels and transferred onto NC membranes (Millipore, Bedford, MA, USA). Then, we used 8% nonfat milk to block nonspecific sites for 1 hour at room temperature. The membranes were then incubated with primary antibodies against vascular endothelial growth factor (VEGF; Servicebio, Wuhan, China), TGFBR1 (Abcam, Cambridge, UK), ID4 (Santa Cruz, Dallas, Texas, USA), C-C motif chemokine ligand 2 (CCL2; Huabio, Hangzhou, China), Smad2 (Santa Cruz, Dallas, Texas, USA), pSmad2 (CST, Danvers, Massachusetts, USA), Smad3 (CST, Danvers, Massachusetts, USA), pSmad3 (CST, Danvers, Massachusetts, USA) and GAPDH (CST, Danvers, Massachusetts, USA) at 4°C overnight. The membranes were washed with TBST 3 times for 10 minutes each and then incubated with a horseradish peroxidase-conjugated goat anti-rabbit or goat anti-mouse IgG secondary antibody (CST, Danvers, Massachusetts, USA) at room temperature for 2 hours according to the species of primary antibody. Finally, the ECL Detection Kit (NCM Biotech, Suzhou, China) was used for detection and photography. Images of protein bands were analyzed using ImageJ software.

### Dual-luciferase reporter gene assay

The 500 bp UTRs of TGFBR1 and ID4 containing the predicted binding sites or mutant predicted binding sites of miR-210-3p were subcloned downstream in the pmirGLO miReport vector. We cotransfected the vector and miR-210-3p mimic or scrambled negative control with Lipofectamine 3000 reagent (Invitrogen, Carlsbad, CA, USA) according to the manufacturer’s instructions. Forty-eight hours after transfection, we lysed the cells and measured firefly and Renilla luciferase activity with the Dual-Luciferase Reporter Assay System (Solarbio, Peking, China).

### Real-time quantitative polymerase chain reaction (RT–qPCR)

Total RNA was extracted from each sample using TRIzol reagent (Thermo, Waltham, Massachusetts, USA), separated using chloroform and precipitated with isopropanol according to the manufacturer’s instructions. Then, reverse transcription was performed using the PrimeScript RT Master Mix Kit (Takara, Tokyo, Japan) to acquire cDNAs. RT–qPCR was performed in a 20 µl reaction system using TB Green Premix Ex Taq (Takara, Tokyo, Japan) according to the manufacturer’s instructions. miR-210-3p and U6 were reverse-transcribed using Bulge-Loop miRNA qRT–PCR Primers (Ribobio, Guangzhou, China). GAPDH and U6 were used as internal references for mRNA and miRNA quantification, respectively. The primer sequences are listed in **Table S2**. Relative gene expression was quantified using the 2^−ΔΔCT^ method.

### Animal experiments

Animal experiments were performed according to the protocol approved by the Department Laboratory Animal Science of Fudan University. We used 6-week-old C57BL/6 mice and assigned 6 mice to each group. All C57BL/6 mice other than those in the control and sham groups received intra-articular (i.a.) or intravenous (i.v.) injection of AAV-control or miR-210-3p/TIE2/AAV 3 weeks before ACLT. For the i.a. injection, the mice were immobilized in the supine position, the left hindlimb was straightened, and the knee joint and surrounding hair were shaved. We used an insulin syringe and vertically injected 10 µl of AAV (1.5×10^12^ v.g./mL) into the knee joint. In addition, 100 µl of AAV (1.5×10^12^ v.g./mL) were used for the i.v. injection. For ACLT, C57BL/6 mice were anesthetized with 0.3% sodium pentobarbital. A 0.5 cm longitudinal incision was made along the left knee joint of the mice. The fascia above the patellar ligament was bluntly separated. A longitudinal incision was made at the medial side of the patellar ligament to open the joint cavity. Then, we pulled the patellar ligament laterally to expose the joint cavity. After blunt separation of the infrapatellar fat pad, we transected the anterior cruciate ligament. Notably, we did not transect the anterior cruciate ligament of the sham group at this step. Then, the joint cavity was washed with saline, and the patellar ligament, fascia and skin were stitched with absorbable sutures after repositioning the ligament.

### *In situ* hybridization (ISH)

ISH staining for miR-210-3p in subchondral bone of normal mice and mice was performed 4 weeks after ACLT. The miR-210-3p probes and ISH kits were purchased from Servicebio (Wuhan, China) and used according to the manufacturer’s instructions. Briefly, paraffin sections of mouse knees were subjected to dewaxing, repair and prehybridization. For hybridization, 50 μL of the probe-containing hybridization solution were added to cover the tissue, and samples were incubated in a humid chamber at 37°C overnight. After the incubation, sections were washed with 37°C prewarmed 2×SSC solution once for 10 minutes and 37°C prewarmed 1×SSC solution twice for 5 minutes each. DAPI was used for nuclear staining. Images were captured using a fluorescence microscope.

### Histology and immunohistochemical analysis

We harvested the left hind knees of mice after euthanasia at 2 or 4 weeks after ACLT and fixed them with 4% formaldehyde for 48 hours. Then, the knees were immersed in 1.5 M EDTA for 1-1.5 weeks for decalcification. Following dehydration in a gradient of alcohol solutions, the knees were embedded in OCT compound for sectioning (6 μm). For safranin-O & fast green staining, sections were dewaxed in water and immersed in fast green staining solution for 6 minutes. After washing with distilled water for 1 minute, the sections were immersed in safranin O solution for 4 minutes, rinsed with distilled water for 1 minute and differentiated in glacial acetic acid for 1 minute. The sections were sealed with neutral resin after dehydration. For immunohistochemical staining, sections were dewaxed in water and repaired in boiled antigen retrieval solution for 8 minutes. After blocking with a 3% BSA/PBS solution for 1 hour at room temperature, sections were incubated with anti-endomucin (Emcn; Santa Cruz, Dallas, Texas, USA), anti-matrix metalloproteinase 13 (MMP13; Abcam, Cambridge, UK) and anti- Runt-related transcription factor 2 (RUNX2; Servicebio, Wuhan, China) antibodies, followed by fluorescent dye-conjugated secondary antibodies (Abcam, Cambridge, UK) for fluorescence imaging and HRP-conjugated secondary antibodies (Servicebio, Wuhan, China) for DAB staining.

### Flow cytometry

We harvested inguinal lymph nodes at 2 weeks after ACLT and digested the lymph nodes with a mixture of type II collagenase (Yeasen, Shanghai, China) and DNase I (Yeasen, Shanghai, China) for 20 min at room temperature. The digest was filtered through a 70 μm cell strainer (Miltenyi, Germany) and then washed with 1× PBS. For staining of cytokine interleukin 17a (IL-17a), cells were stimulated with the leucocyte activation cocktail BD Pharmingen (BD Biosciences, Franklin Lakes, New Jersey, U.S.) for 4 hours at 37°C. Then, the cells were incubated with antibodies against surface markers on ice for 30 minutes in the dark. After fixation and permeabilization with a BD CytoFix/CytoPerm Kit (BD Biosciences, Franklin Lakes, New Jersey, U.S.), cells were stained with IL-17a fluorescent antibodies on ice for an additional 30 minutes in the dark. The T cell panel included the following antibodies: Zombie NTR Fixable Viability APC-Cy7 (Biolegend, San Diego, California, U.S), CD25 PE-Cy7 (Biolegend), CD3 FITC (Biolegend) and IL-17a PE (Biolegend). Analyses were performed using FlowJo software.

### MicroCT analysis

The isolated knee joints were fixed overnight with 4% formaldehyde and analyzed using microCT (Sky-scan 1174, Bruker MicroCT) (voltage, 65 kVp; current, 153 μA; and resolution, 9 μm/pixel). We used image reconstruction software (NRecon v1.6, Bruker), data analysis software (CTAn v1.9, Bruker), and 3-dimensional model visualization software (μCTVol v2.0, Bruker) to analyze the parameters of the tibia subchondral bone. The whole subchondral bone medial compartment was the region of interest, and 100 consecutive images from the medial tibial plateau were used for 3-dimensional reconstruction and analysis. The bone mineral density BMD and bone volume/tissue volume (BV/TV) were measured in three-dimensional structures.

### CatWalk analysis

The CatWalk gait analysis system (Noldus Information Technology) was used to measure the disability of mice in this study. Mice were placed individually in the walkway and allowed to walk freely. When the mouse traversed from one side of the walkway to the other, a high-speed color video camera recorded mouse movements and footprints simultaneously. The software automatically identified all contacted areas and assigned them to the respective paws. The durations and left hind limb duty cycle were analyzed.

### Statistical analysis

We performed statistical analyses using GraphPad Prism 9 software. Numerical data are presented as the mean ± SD. Unpaired two-tailed Student’s t test was used to compare the results from two groups, and one-way ANOVA was used to compare variables between more than two groups. Statistical significance was defined as *P < 0.05 and **P < 0.01.

## Results

### miR-210-3p expression is negatively related to aberrant subchondral angiogenesis in OA

**Figure 1:**
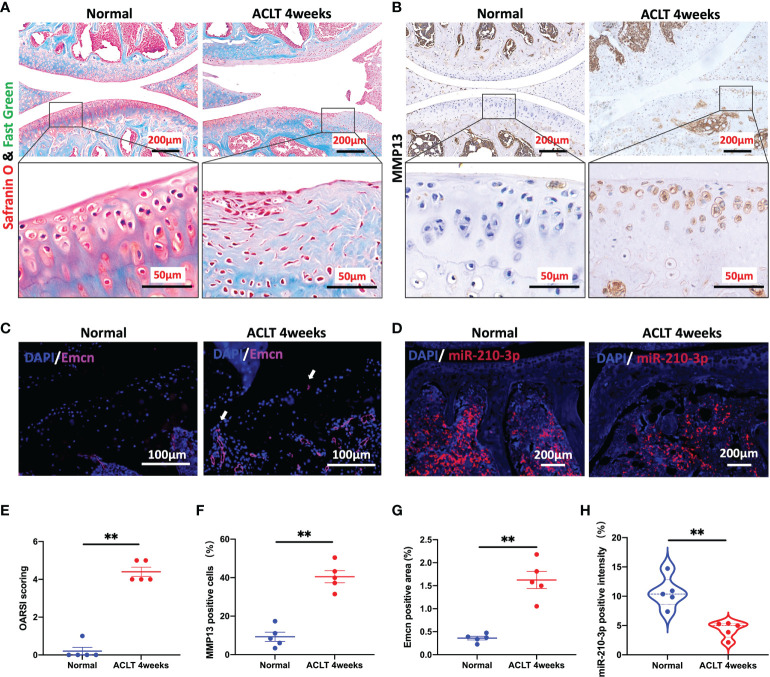
miR-210-3p expression is negatively related to aberrant subchondral angiogenesis in OA. Ten-week-old C57BL/6 mice underwent ACLT. Knee joints were harvested at 4 weeks after surgery. *n* = 5 mice per group. **(A)** Safranin O–fast green staining for the knee joint (sagittal view). Scale bar: 200 μm (top), 50 μm (bottom). **(B)** Immunohistochemical staining for MMP13 in the joint (top) and cartilage (bottom) of normal mice and ACLT for 4 weeks mice. Scale bar: 200 μm (top), 50 μm (bottom). **(C)** Immunofluorescence staining for Emcn (red) and DAPI (blue) in the subchondral bone of tibia. Vessels invading the cartilage are indicated by white arrows. Scale bars: 100 μm. **(D)** ISH staining for miR-210-3p (red) and DAPI (blue) in the subchondral bone of tibia. Scale bars: 200 μm. **(E)** Calculation of OARSI scores. **(F)** Quantification of the number of MMP13-positive cells in cartilage. **(G)** Quantification of the positive area of Emcn staining. **(H)** Quantification of the intensity of miR-210-3p staining in subchondral bone. **P < 0.01. All data are presented as the means ± standard deviations. Statistical significance was determined using unpaired, 2-tailed Student’s *t* test.

We applied ACLT to induce OA in mice as a method to verify the potential correlation between miR-210-3p expression and aberrant vascularization of subchondral bone. At 4 weeks after ACLT, the cartilage was significantly degenerated in ACLT mice, as shown by safranin-O & fast green staining (**Figure 1A**), and the Osteoarthritis Research Society International (OARSI) scores were also significantly increased compared with those of control mice (**Figure 1E**). Meanwhile, the expression of MMP13, a molecule directly involved in cartilage degradation, was significantly increased in the cartilage of ACLT mice compared with control mice (**Figures 1B, F**). Next, we stained for Emcn, a specific marker of capillaries and sinusoids in the metaphysis and diaphysis (29–31), to assess the vasculature in the subchondral bone of mouse knees using immunohistochemical staining. We observed abnormally proliferating Emcn^+^ blood vessels in subchondral bone, and some vessels even invaded the cartilage through the tidemark in ACLT mice (**Figures 1C, G**). We next validated the expression of miR-210-3p in subchondral bone by performing an ISH assay. The expression of miR-210-3p was significantly decreased in subchondral bone of ACLT mice compared with control mice (**Figures 1D, H**). Based on these findings, we speculated that miR-210-3p may be involved in the abnormal vascularization of subchondral bone during the development of OA.

### miR-210-3p regulates the angiogenic ability of HUVECs *in vitro* under hypoxia

**Figure 2:**
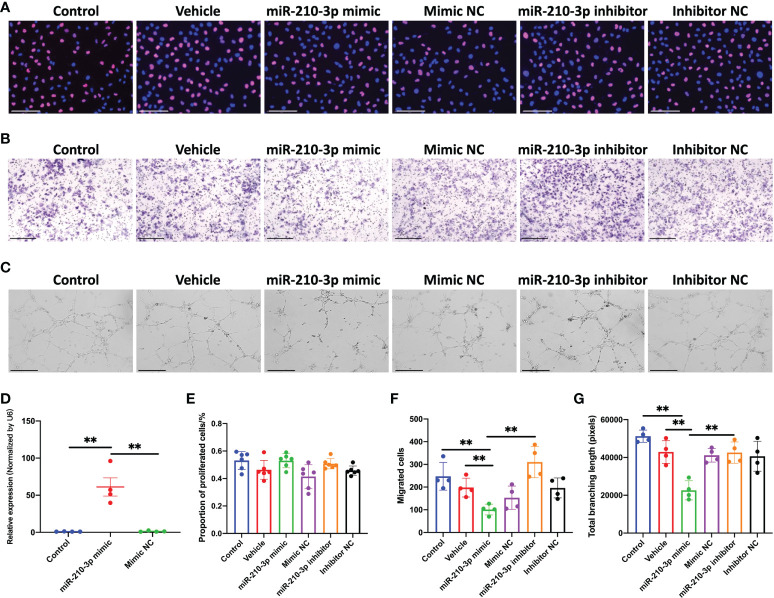
miR-210-3p affects the angiogenic ability of HUVECs in vitro under hypoxia. **(A)** EdU staining for control HUVECs and HUVECs transfected with vehicle, miR-210-3p mimic, mimic NC, miR-210-3p inhibitor or inhibitor NC and cultured under hypoxia (1% O_2_). Scale bars: 150 μm. **(B)** Transwell assay of control HUVECs and HUVECs transfected with vehicle, miR-210-3p mimic, mimic NC, miR-210-3p inhibitor or inhibitor NC and cultured under hypoxia (1% O_2_); Scale bars: 300 μm. **(C)** Representative images of tube formation by control HUVECs and HUVECs transfected with vehicle, miR-210-3p mimic, mimic NC, miR-210-3p inhibitor or inhibitor NC. Scale bars: 275 μm. **(D)** Quantification of miR-210-3p expression levels in control HUVECs and HUVECs transfected with miR-210-3p mimic or mimic NC using RT–qPCR. **(E)** Quantification of proportion of proliferated cells. **(F)** Quantification of the number of migrated cells. **(G)** Quantification of the total branching length of each group. **P < 0.01. NC, negative control. All data are presented as the means ± standard deviations. Statistical significance was determined using unpaired, 2-tailed Student’s *t* test.

We transfected the miR-210-3p mimic or inhibitor into HUVECs *in vitro* to investigate the role of miR-210-3p in aberrant angiogenesis of subchondral bone in OA. The miR-210-3p level was substantially increased after the miR-210-3p mimic transfection, as evidenced by the qPCR results (**Figure 2D**). Briefly, proliferation, migration and tube formation were assessed as measures of the angiogenic ability of ECs. Notably, we found that miR-210-3p had no effect on the angiogenesis ability of HUVECs under normoxic conditions (**Figure S1**). Then, we evaluated the effect of miR-210-3p on the angiogenic ability of HUVECs grown in the presence of 1% oxygen. Our results revealed that miR-210-3p did not affect the proliferation of HUVECs (**Figures 2A, F**) but significantly repressed the migration (**Figures 2B, E**) and tube formation (**Figures 2C, G**) of HUVECs under hypoxic conditions, implying that miR-210-3p may play an important role in EC migration from normoxic to hypoxic regions *in vivo*.

### RNA sequencing of miR-210-3p-overexpressing HUVECs and miR-210-3p target prediction

**Figure 3:**
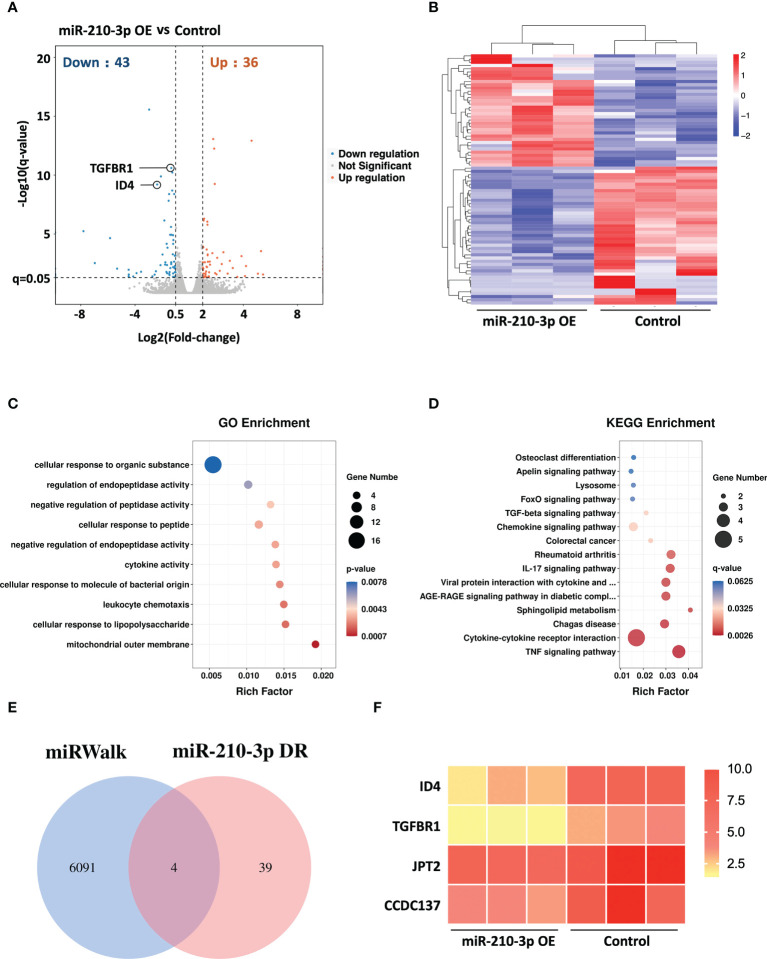
RNA sequencing of miR-210-3p-OE HUVECs and miR-210-3p target prediction. RNA sequencing of miR-210-3p-OE HUVECs compared to the control group under hypoxia (n = 3). **(A)** The differential gene expression analysis showed that miR-210-3p overexpression downregulated 43 genes and upregulated 36 genes (FC > 2, q < 0.05). **(B)** Heatmap of the differentially expressed genes. **(C, D)** KEGG and GO enrichment analyses of genes that were differentially expressed in miR-210-3p-OE cells. **(E)** Venn diagram of miR-210-3p target genes predicted by the miRWalk database intersected with genes downregulated upon miR-210-3p overexpression (FC > 2, q < 0.05). **(F)** Heatmap of the 4 intersecting genes.

We performed RNA sequencing of miR-210-3p mimic-transfected and control HUVECs under hypoxic conditions to evaluate the transcriptional changes in the miR-210-3p-overexpressing (OE) HUVECs. The Benjamini-Hochberg false discovery rate (FDR) method was applied to obtain FDR-adjusted p-values (q-values). Based on our results, forced miR-210-3p OE downregulated 43 genes and upregulated 36 genes (FC > 2, q< 0.05) (**Figure 3A**). These differentially expressed genes were also displayed in a heatmap (**Figure 3B**). The Gene Ontology (GO) enrichment results showed that miR-210-3p mainly affected the process of cellular responses to external stimulation such as cytokines and peptides, which are critical in the angiogenic behavior of ECs (**Figure 3C**). Furthermore, The Kyoto Encyclopedia of Genes and Genomes (KEGG) enrichment analysis showed that VEGF-independent angiogenic signaling pathways, such as the TGF-β signaling pathway and pathways related to cytokine–cytokine receptor interactions, were significantly enriched in miR-210-3p-OE HUVECs (**Figure 3D**). Subsequently, we used the miRWalk database to predict the target genes of miR-210-3p and intersected them with the genes downregulated in miR-210-3p-OE cells identified by RNA sequencing (**Figures 3E, F**). The results showed 4 overlapping genes. Among these genes, ID4 and TGFBR1 were of particular interest because of their proangiogenic properties. ID4 ranked the highest, and ID4 has been reported to promote EC-related angiogenesis by affecting the stability of chemokine mRNAs. TGFBR1 is an important membrane receptor in the TGF-β signaling pathway that is closely related to the angiogenesis of ECs. Therefore, we sought to verify ID4 and TGFBR1 as target genes of miR-210-3p.

### miR-210-3p directly targets TGFBR1 and ID4

**Figure 4:**
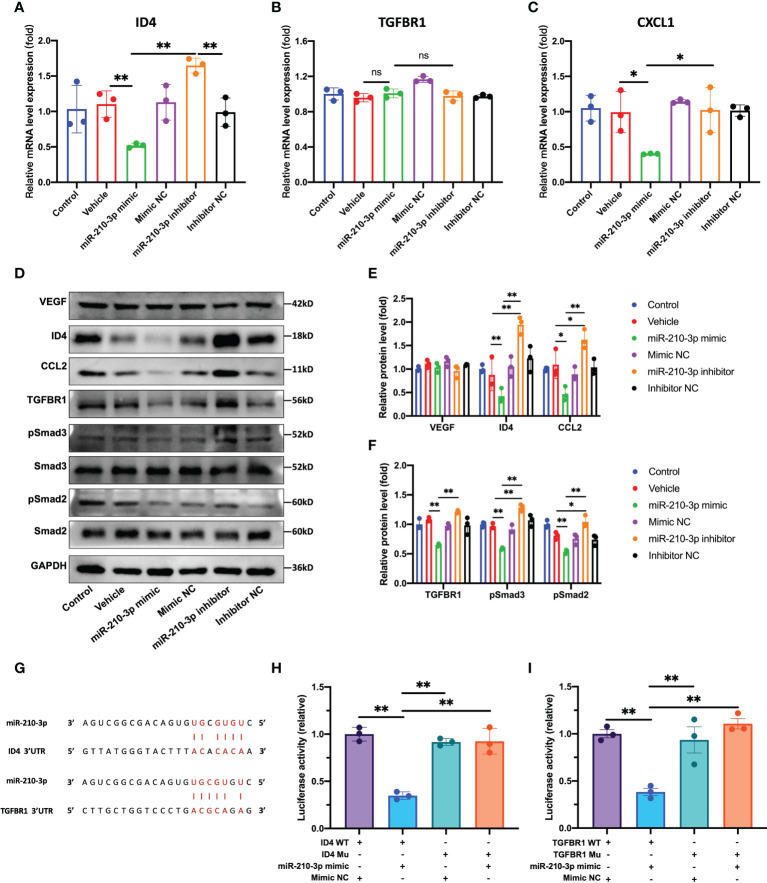
miR-210-3p directly targets TGFBR1 and ID4. **(A–C)** ID4, TGFBR1 and CXCL1 mRNA expression levels quantified by RT–qPCR. **(D–F)** Representative bands showing the levels of the VEGF, ID4, CCL2, TGFBR1, Smad2, pSmad2, Smad3, pSmad3 and GAPDH proteins **(D)**. The relative VEGF, ID4, CCL2 and TGFBR1 protein levels were normalized to the GAPDH level. The relative pSmad2 and pSmad3 protein levels were normalized to those of Smad2 and Smad3, respectively **(E, F)**. **(G)** Predicted binding sites for miR-210-3p in the ID4 and TGFBR1 sequences. **(H, I)** A dual-luciferase reporter assay showed that luciferase activity was significantly inhibited in groups cotransfected with ID4 or TGFBR1 WT vectors and miR-210-3p mimic. **P < 0.01. *P < 0.05. WT, wild-type. Mu, mutant. ns, not significance. All data are presented as the means ± standard deviations. Statistical significance was determined using unpaired, 2-tailed Student’s *t* test.

According to our results, the ID4 mRNA and protein levels were significantly decreased in the miR-210-3p mimic group but were significantly increased in the miR-210-3p inhibitor group compared with the control group (**Figure 4A**), which provided indirect evidence that ID4 is a target gene of miR-210-3p. Consistent with the ID4 expression level, the levels of the proangiogenic chemokines C-X-C motif chemokine ligand 1 (CXCL1) and CCL2 showed similar trends (**Figures 4C, D**), indicating that CXCL1 and CCL2 may be downstream molecules of ID4. Although TGFBR1 mRNA expression level showed no differences among the groups (**Figure 4B**), TGFBR1 protein expression was significantly inhibited by the miR-210-3p mimic and was significantly increased in the miR-210-3p inhibitor group (**Figures 4D, F**). Thus, miR-210-3p mainly inhibits the translation of TGFBR1 instead of degrading the TGFBR1 mRNA. Regarding the activation of downstream molecules in the TGF-β signaling pathway, levels of the phosphorylated forms of Smad2/3 showed a similar trend to TGFBR1 (**Figures 4D, F**), suggesting that miR-210-3p inhibits the activation of the TGF-β signaling pathway by inhibiting TGFBR1 expression. Importantly, the VEGF protein level did not differ among the groups (**Figures 4D, E**), which further illustrated that miR-210-3p regulates the angiogenic ability of ECs through VEGF-independent signaling pathways. We then validated the predicted sites by which miR-210-3p binds to ID4 and TGFBR1 using a dual-luciferase reporter assay (**Figures 4G–I**), providing direct evidence that ID4 and TGFBR1 are target genes of miR-210-3p.

### Knock down of TGFBR1 and ID4 regulates the angiogenic ability of ECs

**Figure 5:**
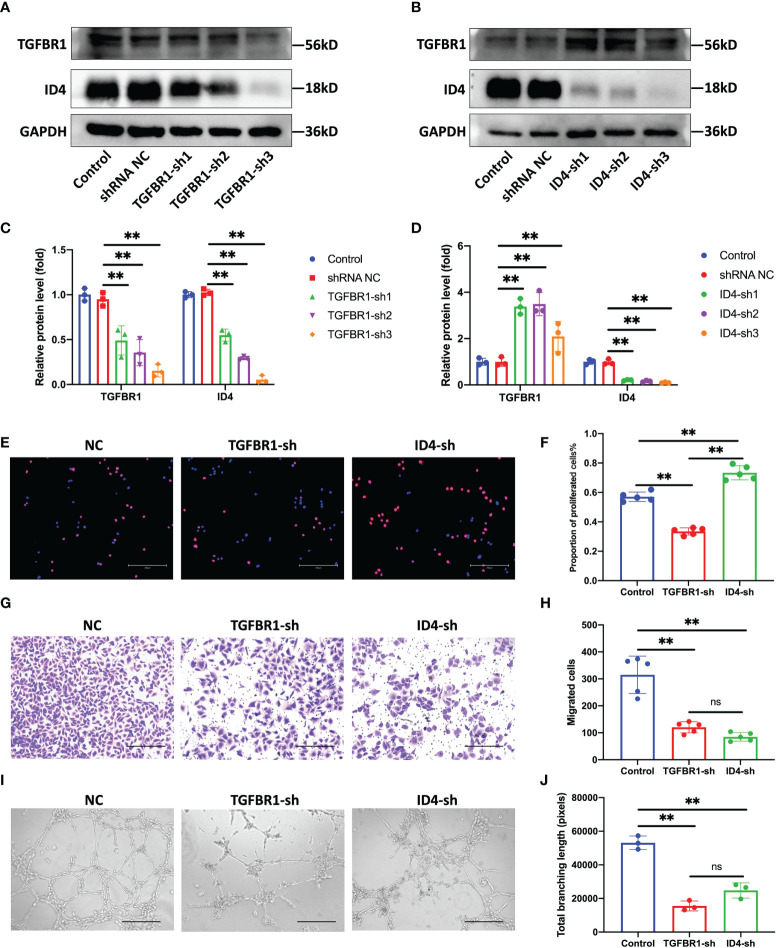
Knock down of TGFBR1 and ID4 regulates the angiogenic ability of ECs. **(A, C)** Representative bands showing the of levels of the TGFBR1 and ID4 protein in control, shRNA NC and TGFBR1-sh1-3 HUVECs **(A)**. The relative TGFBR1 and ID4 protein levels were normalized to those of GAPDH **(C)**. **(B, D)** Representative bands showing the levels of the TGFBR1 and ID4 proteins in control, shRNA NC and ID4-sh1-3 HUVECs **(B)**. The relative TGFBR1 and ID4 protein levels were normalized to those of GAPDH **(D)**. **(E, F)** Representative images of EdU staining and the proportions of proliferating cells in NC, TGFBR1-sh and ID4-sh HUVECs. Scale bar: 300 μm. **(G, H)** Representative images of the transwell assay and the proportions of migrating cells in control, TGFBR1-sh and ID4-sh HUVECs. Scale bar: 300 μm. **(I, J)** Representative images of the tube formation assay and the total branching length of control, TGFBR1-sh and ID4-sh HUVECs. Scale bar: 300 μm. **P < 0.01. ns, not significance. All data are presented as the means ± standard deviation. Statistical significance was determined using unpaired, 2-tailed Student’s t test.

Without interfering with the expression of miR-210-3p, we used shRNAs to knock down the expression of TGFBR1 and ID4 to observe whether this approach was sufficient to inhibit the angiogenesis of ECs. We constructed three shRNA plasmids each for TGFBR1 and ID4 (**Table S1**) and selected one shRNA plasmid each for TGFBR1 and ID4 with satisfactory knockdown (KD) efficiency and no effect on the survival of ECs. Unexpectedly, our results showed that TGFBR1 KD significantly suppressed the expression of ID4 (**Figures 5A, C**), while ID4 KD increased the expression of TGFBR1 (**Figures 5B, D**), suggesting that ID4 is also downstream of TGFBR1 and regulates the expression of TGFBR1 expression through negative feedback. Regarding the angiogenesis of ECs, the proliferation, migration and tube formation ability of the TGFBR1 KD group were significantly decreased. Although the proliferation of the ID4 KD group was increased, the migration and tube formation ability were significantly reduced (**Figures 5E–J**). The effects of TGFBR1 and ID4 KD largely explain the regulation of the angiogenic ability of ECs by miR-210-3p.

### miR-210-3p overexpression induced by AAV attenuates OA progression

**Figure 6:**
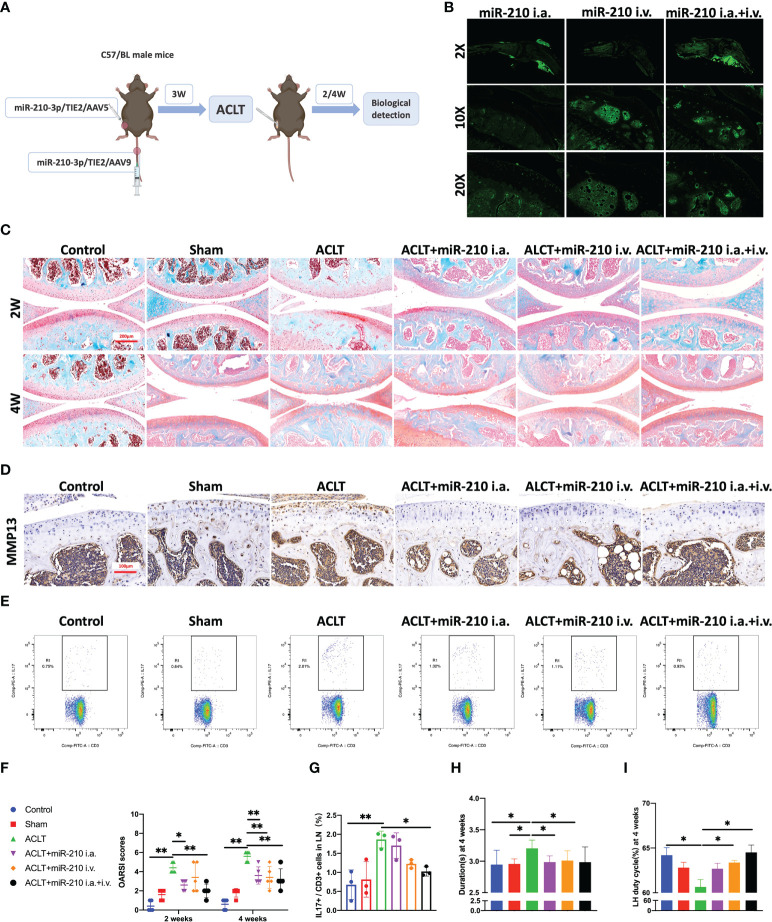
miR-210-3p overexpression induced by AAV attenuates OA progression. **(A)** Design of animal experiments. C57BL/6 mice were i.a. or i.v. injected with miR-210-3p/TIE2/AAV. ACLT was performed to induce posttraumatic OA 3 weeks after AAV administration. Biological and behavioral tests were performed at 2 and 4 weeks after ACLT. **(B)** Representative photos of Zsgreen fluorescence in the affected knees of mice from miR-210-3p/TIE2/AAV i.a., i.v. and i.a.+i.v. injected group. **(C)** Representative images of safranin-O & fast green staining of LH knee joints in each group at 2 weeks and 4 weeks. Scale bar: 200 μm. **(D)** Representative images of immunohistochemical staining for MMP13 in cartilage and subchondral bone of the tibial plateau in each group at 4 weeks. Scale bar: 100 μm. **(E, G)** Flow cytometry images and quantified results for IL-17^+^/CD3^+^ cells in inguinal lymph nodes at 2 weeks after ACLT. **(F)** OARSI scores of LH knees from each group. **(H, I)** Durations and LH duty cycle of each group at 4 weeks after ACLT. **P < 0.01. *P < 0.05. LN, lymph nodes. LH, left hindlimb. All data are presented as the means ± standard deviations. Statistical significance was determined using unpaired, 2-tailed Student’s *t* test.

Based on the results described above, we used miR-210-3p/TIE2/AAV to selectively overexpress miR-210-3p in ECs and observed whether miR-210-3p/TIE2/AAV exerted a therapeutic effect on the OA model. Briefly, we i.a. or i.v. injected miR-210-3p/TIE2/AAV, and ACLT was applied to induce posttraumatic OA 3 weeks after AAV administration. Biological and behavioral tests were performed at 2 and 4 weeks after ACLT (**Figure 6A**). Before ACLT, we assessed Zsgreen fluorescence to determine the efficiency of miR-210-3p/TIE2/AAV infection in each group. Our results illustrated that i.a. administration alone was sufficient to infect the entire knee joint tissue, and i.v. administration induced more obvious infection of blood vessels in subchondral bone, while the i.a.+i.v. administration showed the most pronounced infection intensity (**Figure 6B**). In further therapeutic efficacy assessments, we found that the ACLT+miR-210-3p i.a. group, the ACLT+miR-210-3p i.v. group and the ACLT+miR-210-3p i.a.+i.v. group exhibited alleviated cartilage degradation at 2 and 4 weeks compared with the ACLT group, as evidenced by safranin-O & fast green staining and OARSI scores (**Figures 6C, F**). In addition, the expression of MMP13 in cartilage was also significantly reduced in the three treatment groups compared with the ACLT group (**Figure 6D**). The proportion of IL-17^+^ cells among T lymphocytes (CD3^+^) in inguinal lymph nodes was doubled at 2 weeks after ACLT and significantly decreased in ACLT+miR-210-3p i.a.+i.v. group (**Figures 6E, G**). Regarding behavioral testing, the CatWalk analysis results showed that the time it took for mice to pass through the test channel was significantly prolonged in the ACLT group and significantly decreased in the three treatment groups, especially in the ACLT+miR-210-3p i.v. group and ACLT+miR-210-3p i.a.+i.v. group (**Figure 6H**), while the duty cycle of the left hind limb of mice was significantly reduced in the ACLT group and returned to normal levels in all treatment groups (**Figure 6I**). Based on these results, miR-210-3p/TIE2/AAV had surprising therapeutic efficacy in attenuating OA progression with respect to cartilage protection, immune change as well as symptom relief.

### OA alleviation by miR-210-3p overexpression is attributable to the inhibition of subchondral angiogenesis

**Figure 7:**
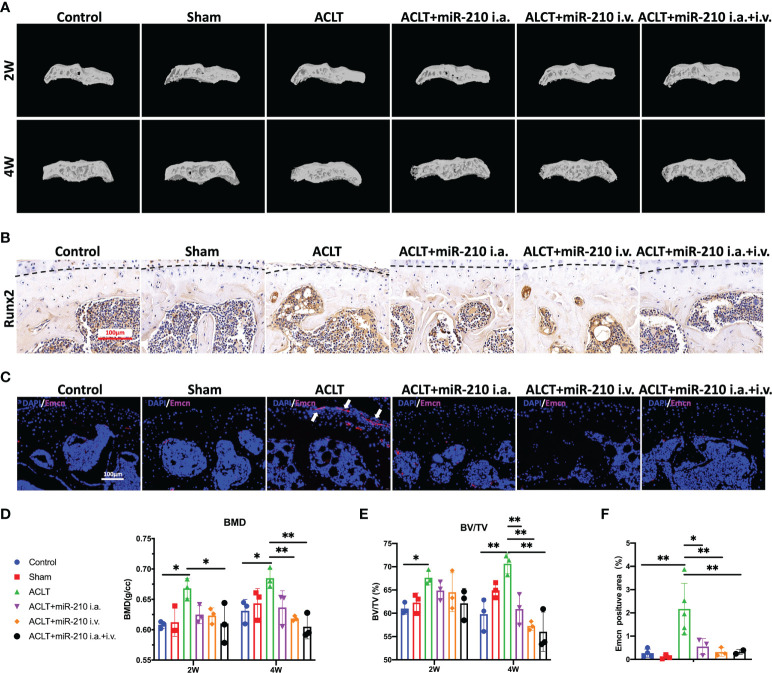
OA alleviation by miR-210-3p overexpression is attributable to the inhibition of subchondral angiogenesis. **(A)** Representative microCT reconstruction images of the tibial plateau of LH knees in each group at 2 weeks and 4 weeks. **(B)** Representative images of immunohistochemical staining for RUNX2 in subchondral bone of the tibial plateau in each group at 4 weeks. Dotted lines: tidemark. Scale bar: 100 μm. **(C)** Representative images of immunofluorescence staining for Emcn (red) and DAPI (blue) in the tibial plateau of LH knees in each group at 4 weeks. Vessels invading the cartilage are indicated by white arrows. Scale bars: 100 μm. **(D, E)** Quantification of the BMD and BV/TV of subchondral bone of the tibial plateau in each group at 2 weeks and 4 weeks. **(F)** Quantification of Emcn positive staining area in each group. **P < 0.01. *P < 0.05. BMD, bone mineral density. BV/TV, bone volume/tissue volume. All data are presented as the means ± standard deviations. Statistical significance was determined using unpaired, 2-tailed Student’s *t* test.

We next assessed subchondral bone remodeling and angiogenesis in each group. The microCT results showed that subchondral bone sclerosis occurred in the ACLT group at 2 weeks and was more severe at 4 weeks, which was manifested as higher intensities in the reconstructed 3D images (**Figure 7A**), increased bone mineral density (BMD) (**Figure 7D**) and increased relative bone volume fractions (**Figure 7E**). However, subchondral bone sclerosis was significantly relieved in the treatment groups, especially in the ACLT+miR-210-3p i.a.+i.v. group. Meanwhile, the expression of RUNX2, a key transcription factor associated with osteoblast differentiation, was significantly increased in subchondral bone of the ACLT group, with a positive zone adjacent to the tidemark (dotted lines), and was significantly decreased in the treatment groups (**Figure 7B**). Notably, Emcn^+^ blood vessels in subchondral bone were abundant and invaded the cartilage layer at 4 weeks after ACLT, as evident from Emcn staining (**Figures 7C, F**). Impressively, the growth of abnormal Emcn^+^ blood vessels in subchondral bone was effectively inhibited in ACLT+miR-210-3p i.a. group and ACLT+miR-210-3p i.v. group and was particularly reduced in the ACLT+miR-210-3p i.a.+i.v. group. In conclusion, gene therapy with miR-210-3p/TIE2/AAV significantly alleviated the process of OA by inhibiting angiogenesis in subchondral bone.

## Discussion

As subchondral bone serves as mechanical support and a dominant source of nutrition for cartilage, alterations in the subchondral bone microenvironment are intimately involved in the progression of OA. These alterations are caused by biological uncoupling and coupling interactions among osteocytes, osteoblasts (OBs), osteoclasts (OCs), ECs and sensory neurons in subchondral bone (15, 32). In the early stage of OA, the balance of subchondral bone remodeling is skewed toward bone resorption in response to altered mechanical stress and inflammation. An increased ratio of receptor activator of nuclear factor κB ligand (RANKL)/osteoprotegerin (OPG) in osteocytes and excessive levels of interleukin (IL)-6, prostaglandin E2 (PGE2) and MMPs produced by OBs promote osteoclastogenesis (33–36). Subsequently, OCs produce proangiogenic cytokines, such as platelet-derived growth factor-BB (PDGF-BB), VEGF and TGF-β, and promote excessive subchondral neurovascularization and bone formation, causing worsening of joint pain and progressive OA (37, 38). We found that miR-210-3p deficiency is responsible for angiogenesis in subchondral bone in an OA model, and selective overexpression of miR-210-3p in ECs effectively attenuated OA progression, as evidenced by cartilage protection, immune changes and improved mobility. In our opinion, the improvement in mobility is a direct reflection of the treatment effect on OA. We found that the mobility of the treatment groups was almost indistinguishable from that of normal mice, which is fairly rare in previous studies (16, 37), providing evidence that miR-210-3p is an effective treatment target for OA.

A substantial number of epigenetic variations and the expression of noncoding RNAs, such as miRNAs, circular RNAs and long noncoding RNAs, are known to dynamically regulate changes in gene expression in normal and OA articular joints (5, 39–43). However, recent studies have mainly focused on functional noncoding RNAs in cartilage and assessed the efficacy of treatments targeting chondrocytes (44–47), and few reports of noncoding RNAs associated with pathological changes in subchondral bone, including subchondral angiogenesis, in OA are available. We found that miR-210-3p expression is significantly downregulated in subchondral bone in an OA model in parallel with aberrant subchondral angiogenesis. Indeed, miR-210-3p is widely considered a proangiogenic miRNA involved in ischemia–reperfusion diseases and cancer (48–50). However, we found that selective miR-210-3p overexpression in ECs induced antiangiogenic effects *in vitro* and *in vivo*. This discrepancy may be due to the differences in the biological effects of different miR-210-3p levels on tissues and ECs. Germana Zaccagnini et al. reported that macrophages with elevated miR-210-3p levels in samples with mismatched miR-210-3p levels in ischemic tissue display dysfunctional angiogenesis, leading to impaired tissue repair (51). This finding further illustrates the heterogeneity of miR-210-3p function in cells and tissues. Consistent with our study, some reports indicate that miR-210-3p inhibits EC function and regulates vascular remodeling in pulmonary hypertension (52, 53) and preeclampsia (28). In summary, we provide the first evidence that miR-210-3p may be exploited as an effective target to inhibit aberrant subchondral angiogenesis in OA. However, we still must explore the molecular mechanism underlying miR-210-3p deficiency in subchondral bone in OA and its effects on processes other than angiogenesis in the subchondral microenvironment in future studies.

AAVs are small (≈25 nm in diameter), nonenveloped, single‐stranded DNA (ssDNA) viruses that serve as promising vectors for gene therapy in animal experiments and clinical trials (54–59). In this study, we applied AAV vectors to overexpress miR-210-3p in subchondral bone vascular ECs. However, more than ten AAV serotypes have been isolated from human and nonhuman primate tissues, and different serotypes have preferences for distinct cells and tissues (60). For i.a. injection, we selected AAV5 for its cartilage permeability and affinity for blood vessels (61–64). For i.v. injection, we chose AAV9 for its extensive ability to invade the circulatory system and affinity for bone tissue (65). In particular, we added TIE2, an endothelial cell-specific promoter, upstream of the miR-210-3p sequence to specifically overexpress miR-210-3p in vascular ECs. Finally, our study showed that miR-210-3p/TIE2/AAV effectively infected subchondral vascular and inhibited aberrant subchondral bone angiogenesis *in vivo*. Apart from its cartilage-protecting effect, miR-210-3p also showed strong inhibition of subchondral bone sclerosis, similar to therapies targeting PDGF-BB (37) or prostaglandin E receptor 4 (EP4) (34) in OCs. However, the group that received i.a. and i.v. injection of miR-210-3p/TIE2/AAV exhibited a reduced BMD of subchondral bone compared with that of the control group, indicating that high levels of miR-210-3p might impair the subchondral vasculature and result in decreased bone formation.

During the development of OA, the inflammatory environment together with complex multicellular interactions affects subchondral angiogenesis, and the molecular mechanisms remain to be elucidated. In the cartilage layer, hypertrophic chondrocytes express high levels of VEGF (66), and cartilage with high mechanical stress secretes excess TGF-β (67). These molecules further promote vascular invasion of cartilage and disrupt cartilage homeostasis during OA development. In OA subchondral bone, TGF-β1 produced by OBs is mainly responsible for subchondral angiogenesis in early OA (11). Our study showed that miR-210-3p inhibits the angiogenic ability of ECs by directly inhibiting the translation of the TGFBR1 mRNA and subsequently affecting Smad2/Smad3 phosphorylation under hypoxia. In addition, we also found that miR-210-3p degrades the ID4 mRNA in ECs. ID4 is a member of a protein family that functions as a negative regulator of helix-loop-helix transcription factors. Giulia Fontemaggi et al. reported that ID4 binds and stabilizes mRNAs encoding the proangiogenic factors IL8 and CXCL1 to promote neuroangiogenesis (68). In our study, we found that ID4 may promote angiogenesis by stabilizing the CXCL1 and CCL2 mRNAs. Interestingly, we identified ID4 as a possible downstream molecule of TGFBR1 that regulates TGFBR1 through negative feedback, producing a strong inhibitory effect on cells overexpressing miR-210-3p. In summary, we verified that miR-210-3p modulates TGFBR1 and ID4 expression and attenuates OA progression by inhibiting subchondral angiogenesis, providing a new therapeutic target for OA treatment.

Nonetheless, this study still has some limitations. First, we did not assess changes in the expression of miR-210-3p in normal and OA human knee joints. Furthermore, the therapeutic effect of miR-210-3p on human OA remains to be further validated. Second, we tested the treatment efficacy of miR-210-3p at 2 weeks and 4 weeks after ACLT in mice, but its long-term therapeutic effects and side effects related to the cardiovascular system and vital organs still require further investigation. Finally, this study only preliminarily suggested that ID4 is downstream of TGFBR1, and we are working to elucidate the specific underlying molecular mechanism.

## Conclusion

In conclusion, our study provides the first evidence that miR-210-3p inhibits the angiogenic ability of ECs by directly targeting TGFBR1 and ID4, and the miR-210-3p/TGFBR1/ID4 axis in subchondral ECs modulates the progression of OA *via* subchondral angiogenesis, which might represent a potential target for OA therapy.

## Data availability statement

The RNA-seq data of effect of overexpression of miR-210-3p on gene expression of human umbilical vein endothelial cells in hypoxic conditions is available in the GEO database with accession number GSE214170.

## Ethics statement

The animal study was reviewed and approved by The Department Laboratory Animal Science Fudan University.

## Author contributions

HT, WZ, LC, CG, and DM developed the concept for the study. HT and LC designed the research plan. HT and WZ performed the experiments and analyzed data. HT and WZ wrote and edited the manuscript. LC, JZ, and JL revised the manuscript. All authors read and approved the final version of the manuscript.

## Funding

This study was supported by the National Key Research and Development Program of China (Grant. No. 2021YFC270101), the Science and Technology Commission of Shanghai Municipality (Grant. No. 22ZR1410800) and the National Natural Science Foundation of China (Grant. No.8167090095).

## Conflict of interest

The authors declare that the research was conducted in the absence of any commercial or financial relationships that could be construed as a potential conflict of interest.

## Publisher’s note

All claims expressed in this article are solely those of the authors and do not necessarily represent those of their affiliated organizations, or those of the publisher, the editors and the reviewers. Any product that may be evaluated in this article, or claim that may be made by its manufacturer, is not guaranteed or endorsed by the publisher.

## Abbreviations

OA, osteoarthritis; ECs, endothelial cells; TGFBR1, transforming growth factor beta receptor 1; ID4, DNA-binding inhibitor 4; AAV, adeno-associated virus; TIE2, tyrosine kinase with immunoglobulin-like and EGF-like domains 2; ACLT, anterior cruciate ligament transection; IGF2, insulin-like growth factor 2; ECs, endothelial cells; OARSI, Osteoarthritis Research Society International; MMP13, metalloproteinase 13; Emcn, endomucin; ISH, *in situ* hybridization; HUVECs, human umbilical vein endothelial cells; RT-qPCR, real-time quantitative polymerase chain reaction; FDR, false discovery rate; GO, gene ontology; KEGG, Kyoto Encyclopedia of Genes and Genomes; VEGF, vascular endothelial growth factor; CXCL1, C-X-C motif chemokine ligand 1; CCL2, C-C motif chemokine ligand 2; IL-17, interleukin 17; BMD, bone mineral density; RUNX2, runt-related transcription factor 2; OBs, osteoblasts; OC, osteoclasts; RANKL, receptor activator of nuclear factor κB ligand; OPG, osteoprotegerin; PGE2, prostaglandin E2; ssDNA, single‐stranded DNA; PDGF-BB, platelet-derived growth factor-BB; EP4, prostaglandin E receptor 4.
