# Supplementary material for: miR-210-3p protects against osteoarthritis through inhibiting subchondral angiogenesis by targeting the expression of TGFBR1 and ID4
Source: Front Immunol. 2022 Sep 29;13:982278. doi: 10.3389/fimmu.2022.982278 (PMC9575949; doi:10.3389/fimmu.2022.982278)
Supplement: Supplementary file 1 [file DataSheet_1.docx]

**
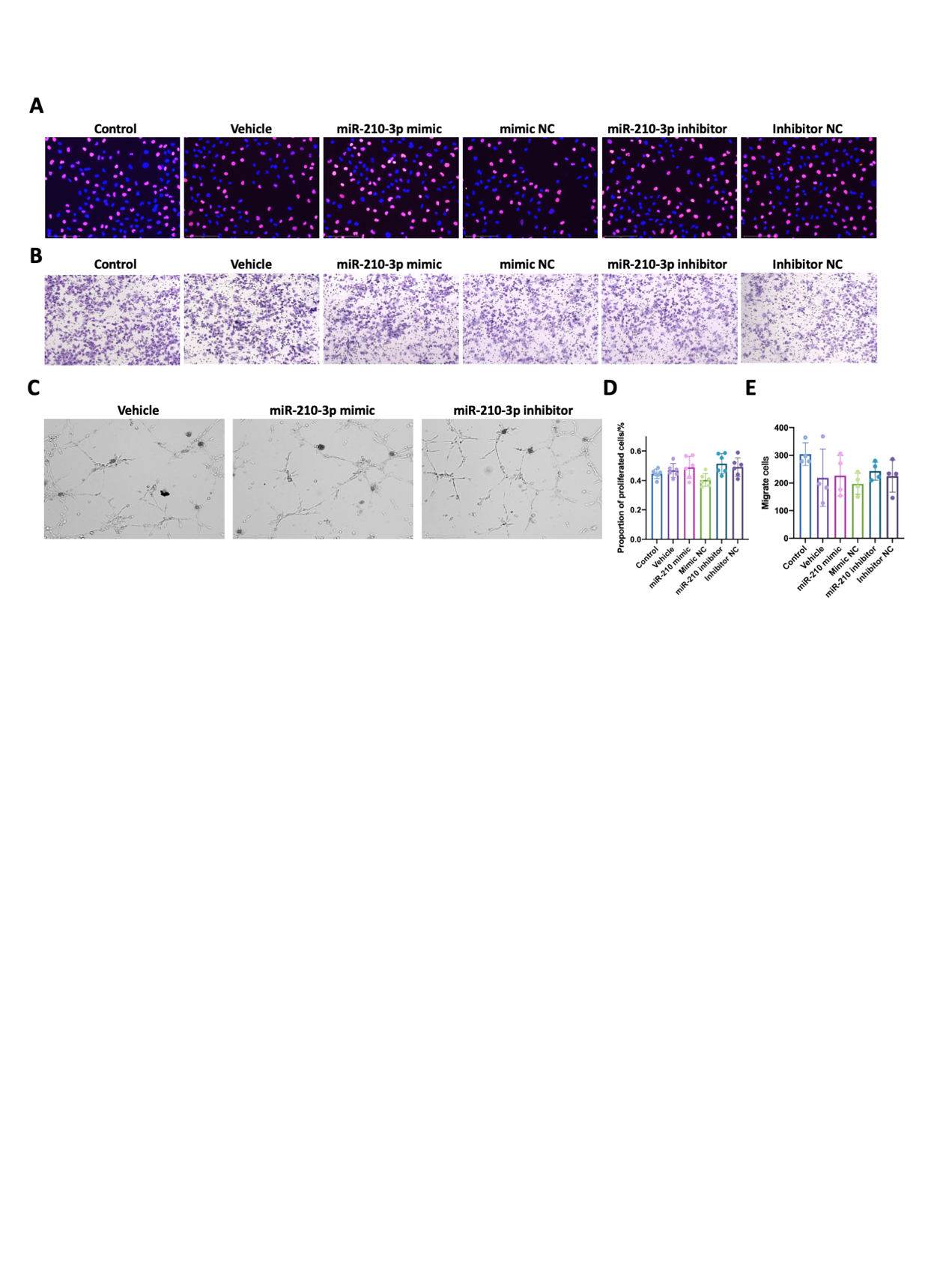
**

**Figure S1. miR-210-3p doesn’t inhibit angiogenesis ability of HUVECs under normoxia. (A, D)** EdU staining was not differ between HUVECs transfected with vehicle, miR-210-3p mimic, mimic NC, miR-210-3p inhibitor and inhibitor NC under normoxia. **(B, E)** Transwell results were similar between groups with no significance in quantification of migration cells. **(C)** Tube formation of HUVECs was not differ between groups. All data are shown as means ± standard deviations. Statistical significance was determined by unpaired, 2-tailed Student’s *t* test.

| shRNA NC | Forward | CCGGCAACAAGATGAAGAGCACCAACTCGAGTTGGTGCTCTTCATCTTGTTGTTTTTG |
| --- | --- | --- |
|  | Reverse | AATTCAAAAACAACAAGATGAAGAGCACCAACTCGAGTTGGTGCTCTTCATCTTGTTG |
| TGFBR1-sh1 | Forward | CCGGGCTGGTCTTAACTTTAGGTAACTCGAGTTACCTAAAGTTAAGACCAGCTTTTTG |
|  | Reverse | AATTCAAAAAGCTGGTCTTAACTTTAGGTAACTCGAGTTACCTAAAGTTAAGACCAGC |
| TGFBR1-sh2 | Forward | CCGGCTCATGTTGATGGTCTATATCCTCGAGGATATAGACCATCAACATGAGTTTTTG |
|  | Reverse | AATTCAAAAACTCATGTTGATGGTCTATATCCTCGAGGATATAGACCATCAACATGAG |
| TGFBR1-sh3* | Forward | CCGGCCCTTCATTAGATCGCCCTTTCTCGAGAAAGGGCGATCTAATGAAGGGTTTTTG |
|  | Reverse | AATTCAAAAACCCTTCATTAGATCGCCCTTTCTCGAGAAAGGGCGATCTAATGAAGGG |
| ID4-sh1* | Forward | CCGGCCCAACAAGAAAGTCAGCAAACTCGAGTTTGCTGACTTTCTTGTTGGGTTTTTG |
|  | Reverse | AATTCAAAAACCCAACAAGAAAGTCAGCAAACTCGAGTTTGCTGACTTTCTTGTTGGG |
| ID4-sh2 | Forward | CCGGGCGATATGAACGACTGCTATACTCGAGTATAGCAGTCGTTCATATCGCTTTTTG |
|  | Reverse | AATTCAAAAAGCGATATGAACGACTGCTATACTCGAGTATAGCAGTCGTTCATATCGC |
| ID4-sh3 | Forward | CCGGCGCGGTGAACAAGCAGGGCGACTCGAGTCGCCCTGCTTGTTCACCGCGTTTTTG |
|  | Reverse | AATTCAAAAACGCGGTGAACAAGCAGGGCGACTCGAGTCGCCCTGCTTGTTCACCGCG |

**Table S1. ShRNA sequences of TGFBR1 and ID4.** * TGFBR1-sh3 and ID4-sh1 were used in subsequent experiments.

| Gene | Species | Forward/Reverse | 5’->3’ |
| --- | --- | --- | --- |
| TGFBR1 | Human | Forward | TCAGCTCTGGTTGGTGTCAG |
|  |  | Reverse | ATGTGAAGATGGGCAAGACC |
| ID4 | Human | Forward | CACGTTATCGACTACATCCTGG |
|  |  | Reverse | TGTCGCCCTGCTTGTTCAC |
| GAPDH | Human | Forward | GGAGCGAGATCCCTCCAAAAT |
|  |  | Reverse | GGCTGTTGTCATACTTCTCATGG |
|  |  | Reverse | CCTTATCAAGATGCGAACTCACA |
| CXCL1 | Human | Forward | GCGCCCAAACCGAAGTC |
|  |  | Reverse | TGCAGGATTGAGGCAAGCTT |

**Table S2. Primer sequences of TGFBR1, ID4, GAPDH and CXCL1 in RT-qPCR.**
